# Language Learning Enhanced by Massive Multiple Online Role-Playing Games (MMORPGs) and the Underlying Behavioral and Neural Mechanisms

**DOI:** 10.3389/fnhum.2017.00095

**Published:** 2017-03-02

**Authors:** Yongjun Zhang, Hongwen Song, Xiaoming Liu, Dinghong Tang, Yue-e Chen, Xiaochu Zhang

**Affiliations:** ^1^Center for Biomedical Engineering, School of Information Science and Technology, University of Science and Technology of China Hefei, China; ^2^School of Foreign Languages, Anhui Jianzhu University Hefei, China; ^3^School of Humanities and Social Science, University of Science and Technology of China Hefei, China; ^4^School of Public Affairs, University of Science and Technology of China Hefei, China; ^5^CAS Key Laboratory of Brain Function and Disease, School of Life Science, University of Science and Technology of China Hefei, China; ^6^State Key Laboratory of Brain and Cognitive Science, Institute of Biophysics, Chinese Academy of Sciences Beijing, China; ^7^Center of Medical Physics and Technology, Hefei Institutes of Physical Science, Chinese Academy of Sciences Hefei, China

**Keywords:** Massive Multiple Online Role-Playing Games (MMORPGs), language learning, interaction, reward, behavioral mechanism, neural mechanism

## Abstract

Massive Multiple Online Role-Playing Games (MMORPGs) have increased in popularity among children, juveniles, and adults since MMORPGs’ appearance in this digital age. MMORPGs can be applied to enhancing language learning, which is drawing researchers’ attention from different fields and many studies have validated MMORPGs’ positive effect on language learning. However, there are few studies on the underlying behavioral or neural mechanism of such effect. This paper reviews the educational application of the MMORPGs based on relevant macroscopic and microscopic studies, showing that gamers’ overall language proficiency or some specific language skills can be enhanced by real-time online interaction with peers and game narratives or instructions embedded in the MMORPGs. Mechanisms underlying the educational assistant role of MMORPGs in second language learning are discussed from both behavioral and neural perspectives. We suggest that attentional bias makes gamers/learners allocate more cognitive resources toward task-related stimuli in a controlled or an automatic way. Moreover, with a moderating role played by activation of reward circuit, playing the MMORPGs may strengthen or increase functional connectivity from seed regions such as left anterior insular/frontal operculum (AI/FO) and visual word form area to other language-related brain areas.

## Introduction

Massive Multiplayer Online Role-Playing Games (MMORPGs) are gaining more and more popularity compared to other genres of commercial games. The main feature of MMORPGs is gamers’ purposeful interaction with peers and game-embedded narratives elicited by the game design. The players’ ultimate purpose is to get reward so as to progress through the game hierarchy by undertaking game tasks known as quests, usually with the help of game-based organizations known as guilds. Guild membership offers novices opportunities to get their gaming skills promoted through interaction with more experienced players (Peterson, 2012). Notably, MMORPGs may bring about some negative effects such as excessive playing or gaming addiction (Petry and O’Brien, 2013), and psychiatric comorbidity (Han et al., 2015). However, MMORPGs can also provide players with benefits such as feelings of achievement and sense of community (Sublette and Mullan, 2012), and possibilities for educational use (González-González and Blanco-Izquierdo, 2012).

Applying MMORPGs to foreign language (FL) or second language (L2) learning has become a research focus in that, gamers/learners immersed in MMORPGs learning context are more relaxed and motivated to interact with peers or gaming instructions (Bytheway, 2014), and they outperform those attending traditional classrooms in terms of language skills (Rankin et al., 2009; Suh et al., 2010; Kim et al., 2013). The main affordances of MMORPGs for language learning are the immersive interactive environments and multiple options for players to engage in authentic communication through listening, speaking, reading, and writing in the target language with other interlocutors (Rama et al., 2012). Apart from the commercial MMORPGs, researchers develop some educational MMORPGs to facilitate FL/L2 learning. Such educational MMORPGs are also named serious games, which “include an identifiable teaching presence specifically for improving some aspect of language proficiency” (Thorne et al., 2012a). Serious games’ main objectives are learning and behavior change (Connolly et al., 2012) and can also provide gamers with in-game rewards if they accomplish tasks (Nagle et al., 2014), leading to greater learning motivation and more effective learning relative to traditional tools or approaches (Iten and Petko, 2016). In this paper, we briefly review studies focusing on commercial or serious MMORPGs’ benefit to learning FL/L2 and discuss the potential mechanisms underlying the educational assistant role of MMORPGs in language learning from behavioral and neural perspectives.

## Methods

We searched for literatures on Google Scholar, Web of Science, and ScienceDirect with no date restrictions. Terms used were “massive multiplayer online role^∗^” or “MMORPG^∗^” in combination with “language learning” or “second language” or “FL” or “language teaching.” Since some online games especially 3D online games bear features of MMORPGs, we also used terms like “online game” and “3D online game” in our search. We finally selected the most relevant papers for our review and some studies were identified through checking reference lists of the indexed papers. Available studies were organized in two groups based on their aims: macroscopic studies on MMORPGs’ benefit for gamers/learners’ overall FL/L2 learning and microscopic studies on MMORPGs’ benefit for one or more specific FL/L2 abilities.

## MMORPGs’ Benefit for Gamers’/Learners’ Fl/L2 Learning

In the studies conducted by Rankin et al. (2008), Zheng et al. (2009, 2012), and Rama et al. (2012), MMORPG’s affordance of interaction was found to benefit FL/L2 acquisition or development. Interestingly, Zheng et al. (2009, 2012) found that gamers can realize their heterarchical values while learning English in MMORPG’s interactive context. Peterson (2011, 2012) attached more importance to learners’ attitudes exhibited in MMORPG-based interaction. The former study suggested that the MMORPG-based interaction can lead to learners’ positive feedback, by which language development may be facilitated. The latter study showed that in online linguistic and social interaction, learners adopted polite expressions to build up collaborative relationships, used continuers and requests for assistance to maintain intersubjectivity, and became increasingly positive toward gaming and language learning emerged in gaming. Thus, such interaction can contribute to learners’ sociocultural competence, positive attitudes toward FL learning, and coherence and appropriateness of target language production, all of which are beneficial for FL development. Considering that gamers are involved in both virtual spaces and real world settings, researchers are interested to ascertain whether the language learning-related resources and interactions in and out of the MMORPGs’ context can influence each other or work together to promote the gamers’ language development. In two successive studies, Kongmee et al. (2011, 2012) validated that linguistic knowledge and communicational skills can be transferable between the virtual spaces and real world. Scholz (2015) reached a similar conclusion that if learners are given the opportunity to communicate with other players and experience the game at their own pace, they can transfer linguistic constructions from MMORPGs’ contexts to various non-gaming contexts, so that L2 learning can be developed more effectively. To dig it further, Thorne et al. (2012b) employed semiotic ecology theory to indicate that game-embedded texts, player-to-player interaction, and game-external websites’ resources constitute gamers/learners’ complex semiotic ecologies, which are significant for L2 development.

Comparatively, more studies have examined the effect of MMORPG on enhancing gamers’ some specific FL/L2 abilities. In view of the central place of vocabulary in language learning, some studies have argued that vocabulary learning can be facilitated by gamers’ interaction in playing MMORPGs (Bytheway, 2014; Shahriarpour and Kafi, 2014; Yudintseva, 2015; Zheng et al., 2015). Contrary to these studies, Milton et al. (2012) reached a relatively conservative conclusion that there is little opportunity for lexical growth without teacher’s control in the MMORPG-based learning activities. Other studies have shown that vocabulary acquisition and other skills such as communicative competence (Peterson, 2010), sentence construction (Yang and Hsu, 2013), and reading skills (Dourda et al., 2014) can be developed simultaneously by gamers’ interaction in MMORPG-based instruction. Besides, Huang and Yang (2014) investigated effects of English proficiency and gaming experience on incidental vocabulary acquisition in a MMORPG and found that vocabulary was more noticed by learners with medium gaming experience in gaming requirement condition, and was more perceived by learners with higher English proficiency in flashcard condition. Apart from above-mentioned studies focusing on vocabulary development in playing MMORPGs, many studies have demonstrated the positive effects of MMORPGs on developing basic language skills such as FL listening ability (Hu and Chang, 2007), speaking ability (Lai and Wen, 2012), production of narratives (Colby and Colby, 2008; Neville, 2010, 2015), communicative competence (Wu and Richards, 2012; Berns et al., 2013), and communicative skills, together with learners’ listening, reading, and writing skills (Suh et al., 2010). In addition, Hsu (2015) reported that the MMORPG has long-term effects on developing learners’ incremental intelligence (i.e., accumulated intelligence through hard work) which was significantly related to their performance on standardized language test.

It is indicated that existing studies have mainly explored MMORPGs’ benefit for FL/L2 learning based on MMORPGs’ affordance of interactive function. Specifically, MMORPGs afford gamers opportunities to communicate with peers from the same guild. Such communication requires active negotiation of meaning in FL/L2 among gamers so that their language skills can be developed (Bytheway, 2011; Rama et al., 2012). Meanwhile, gamers also interact with game-embedded narratives or instructions and they may get positive feedback so as to move on if the embedded texts are properly understood. Notably, when comprehending those embedded texts, gamers may frequently ask for their peers’ help (Dourda et al., 2014). Accordingly, some researchers (Thorne, 2008; Peterson, 2012; Sundqvist and Sylvén, 2012) have tried to explain MMORPGs’ role in facilitating language learning from a sociocultural perspective that employs Vygotsky’s zone of proximal development, which is “the distance between the actual developmental level as determined by independent problem solving and the level of potential development as determined through problem solving under adult guidance or in collaboration with more capable peers” (Vygotsky, 1978). They have suggested that FL/L2 learning can be promoted by in-game social interaction, during which less proficient gamers/learners can negotiate meaning with and learn from more capable gamers/learners. This explanation sheds light on the FL/L2 development process in gaming. However, the underlying behavioral and neural mechanisms of MMORPG-based FL/L2 development remain unexplored. Because learners/gamers are more motivated to interact with peers in MMORPGs’ contexts than they are in traditional teaching settings (Peterson, 2011, 2012; Bytheway, 2014; Shahriarpour and Kafi, 2014; Zheng et al., 2015; Howard-Jones and Jay, 2016), to figure out the source of such stronger motivation appears to be fundamental for investigating the behavioral and neural mechanisms under discussion. Evidence has shown that rewarding the gamers for meeting progressively demanding performance levels increased gamers’ intrinsic motivation (Cameron et al., 2001; Pierce et al., 2003). More recent studies have also shown that rewards such as virtual badges have positive effects on increasing learners’ motivation and learning outcomes in serious games (Filsecker and Hickey, 2014), and that gamers may take meta-game reward systems as intrinsically motivating in game contexts (Cruz et al., 2015). Therefore, reward is an essential factor in motivating gamers/learners to get involved in the in-game interaction and should be taken into account when the behavioral and neural mechanisms of MMORPGs’ role in promoting FL/L2 learning are investigated.

## Possible Behavioral Mechanism Underlying MMORPGs’ Educational Role in Language Learning

Recent studies have validated strong reward effects on the allocation of attention (Hickey et al., 2010; Anderson et al., 2011; Anderson and Yantis, 2013; Lucas et al., 2013), and have shown that stimuli associated with reward in both current and past contexts can bias attentional selection (Anderson et al., 2013; Bourgeois et al., 2015). Furthermore, social rewards such as positive expressions can also shape attentional bias (Anderson, 2015). An integrated review conducted by Le Pelley et al. (2016) concluded that reward influences attention to reward-relevant stimuli. These findings provide us with a deeper insight into the potential behavioral mechanism involved in MMORPG-based language learning. In MMORPGs, reward-associated stimuli can range from some certain gaming skills to interaction with game-embedded texts and peers, which can lead to accomplishment of quests and reward procurement. When gamers/learners are engaged in MMORPGs, they may procure both monetary-like reward such as badges or superior equipment and social reward such as compliments from peers, which prompt them to bias attention and allocate more cognitive resources toward all the reward-related cues emerged in either real-time gaming or past gaming behavior. We thus hypothesize that the potential behavioral mechanism may relate closely to learners’ attentional bias toward both gaming process and gamers’ interaction with embedded game texts and other gamers.

Attentional bias has been validated as a behavioral tendency among excessive online gamers, who generally distribute more attention to game-related cues such as words or pictures and increase their emotional processing of those cues (for a review, see Zhang et al., 2016). Most studies reviewed here didn’t filter participants, and thus included excessive gamers, casual gamers, and novice ones. As such it is worth discussing if the casual and novice gamers are also likely to exhibit attentional bias. An event-related potentials study conducted by Thalemann et al. (2007) revealed casual players also distributed more attention to game-related materials than to neutral cues and they might be highly emotionally involved in online gaming. Han et al. (2010) recruited healthy novices and asked them to play a novel online game for 10 days. Activity was elicited in the dorsolateral prefrontal cortex (DLPFC), parahippocampal gyrus, and thalamus by game cues in contrast to neutral cues for all participants. It is DLPFC that has been found to be related with attentional bias in some studies (Luijten et al., 2012; Jacob et al., 2014). Based on these findings, we may cautiously reach a preliminary conclusion that attentional bias may also arise among casual gamers and novices after they are engaged in online games for a certain period.

Since attentional selection can be operated via a volitional top-down mode derived from task demands or an automatic bottom-up mode triggered by salient stimuli (Corbetta and Shulman, 2002; Buschman and Miller, 2007; Shomstein et al., 2010; Lee and Shomstein, 2014), how gamers/learners employ the two different modes to allocate their attentional resources is another issue warranting consideration. Le Pelley et al. (2016) raised the question whether attentional bias to task-relevant stimuli is a top-down (under participants’ control) or a bottom-up (automatic) process, and they suggested that it was premature to define which one takes effect, because existing studies can be explained by either the former or the latter, or a combination of the two. As to the context of MMORPGs, we suppose that the two processes can be adopted in different ratios by different types of players. For novice players, they may more frequently use the top-down process in which they have to strategically control their own gaming behavior and allocate attentional resources to task-related cues in order to make less mistakes, while for the players with higher gaming proficiency, they tend to utilize more of the bottom-up process, because those task-related cues are psychologically more salient for them and their gaming experiences are rich enough to exert an automatic effect on attentional capture.

## Possible Neural Mechanism Underlying MMORPGs’ Educational Role in Language Learning

Language processing depends on a widely distributed brain network, and specific first or second language abilities are proven to be positively related with various functional connectivities (FC) within this language network (Wei et al., 2012; Deng et al., 2015; Chai et al., 2016). Furthermore, similar brain areas can be activated in both language learning and online gaming (Khatibi and Cowie, 2013). We therefore suggest that gamers/learners’ frequent in-game interaction may strengthen or increase their FC associated with language processing. Additionally, in view of the reward effect on gamers’ motivation to interact in FL/L2 (Peterson, 2012; Howard-Jones and Jay, 2016), we further posit that brain reward circuit may play a moderating role in the increased FC within gamers’ brain network.

To date, very few studies have explored the neural mechanism underlying MMORPG’s educational role in language learning. Only one recent study using resting-state functional magnetic resonance imaging (fMRI) investigated an educational MMORPG’s effect on increasing learners’ brain FC responsible for language processing (Hong et al., 2016). This study did not include control groups, which might make its conclusion less robust. Thus, a cohort study design is needed to ensure more tenable results. Additionally, the specific seed regions identified for FC analysis are also worth further discussion. Studies covered in this review have revealed that MMORPGs’ assistant role in FL/L2 learning is realized by games’ affordance of interaction, in which gamers/learners should frequently retrieve appropriate vocabulary from their memory to fulfill their real-time in-game interaction; moreover, they have to continuously and rapidly, in most cases, read the game-embedded texts and peers’ real-time speech scrolling down the screen to move on smoothly. Such opportunities to develop reading and vocabulary skills are favored by MMORPG players (Peterson, 2011). Therefore, lexical retrieval and reading speed, two central aspects of language processing (Chai et al., 2016), seem to be essential in language learning emerged in playing MMORPGs. Lexical retrieval is linked to left anterior insular/frontal operculum (AI/FO; Perani et al., 2003; Damasio et al., 2004; Baldo et al., 2006), and reading speed is associated with visual word form area (VWFA; Gaillard et al., 2006; Nakamura et al., 2012). Thus, left AI/FO and VWFA can be taken as seed regions in the underlying FC. As for the location of other language areas to which the FC is computed from the seed regions, language processing-related areas in the neural substrates of gamers’ attentional bias should be included. The two above-mentioned modes of attentional bias are controlled by two segregated networks of brain areas. The top-down mode recruits superior frontal gyrus (SFG) and intraparietal cortex, while the bottom-up mode recruits inferior frontal gyrus (IFG) and temporoparietal cortex (Corbetta and Shulman, 2002; Lee and Shomstein, 2014). Both the SFG and the IFG are closely related with language processing. The SFG is associated with language organization (Kinoshita et al., 2012), syntactic sequencing (Chan et al., 2013), speech initiation and spontaneity (Fujii et al., 2015), while the IFG relates to sentence comprehension (Friederici et al., 2003), phonological processing (Nixon et al., 2004), and semantic processing (Simard et al., 2013). The IFG and the SFG can be involved in the increased FC within gamers’ brain network.

**FIGURE 1:**
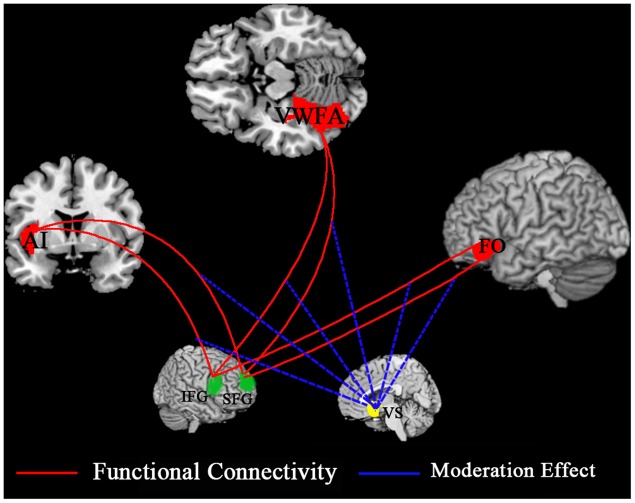
**Potential neural mechanism of Massive Multiple Online Role-Playing Games (MMORPGs’) effect on foreign language (FL)/second language (L2) learning.** AI, anterior insula; FO, frontal operculum; VWFA, visual word form area; IFG, inferior frontal gyrus; SFG, superior frontal gyrus; VS, ventral striatum.

Regarding the identification of areas in the reward circuit, two central nodes involved are ventral striatum (VS) related to reward anticipation and ventromedial prefrontal cortex (vmPFC) related to reward outcome and subjective value (Knutson et al., 2003; Levy and Glimcher, 2012). However, the VS may contribute more to the neural mechanism under discussion, because game behavior associated with reward anticipation processing always takes much more time than reward attainment accompanied by outcome processing does. The potential neural mechanism is shown in **Figure 1**.

## Conclusion and Future Study

To our knowledge, this is the first review centering on both the MMORPGs’ benefits for language learning and discussion of the behavioral and neural mechanisms underlying such benefits. When gamers/learners are immersed in a MMORPG environment, their existing attentional bias or the bias developed in their gaming and learning processes would make them allocate more cognitive resources toward task-related stimuli. Moreover, this reward-guided effect can be realized in a controlled or an automatic way by different types of gamers/learners. Language learning enhancement in playing MMORPGs may be realized by strengthening or increasing the FC from seed regions including the left AI/FO and the VWFA to other language processing-related areas, mainly including the IFG and the SFG. Further, MMORPGs’ effect on the FC can be moderated by the activity of the VS in the brain reward circuit, which warrants further systematic study.

In future studies stroop or dot-probe task can be adopted to examine the existence of attentional bias among gamers/learners whose FL/L2 proficiency get improved after playing MMORPGs. For validation of the proposed neural mechanism, either the resting-state fMRI or task-state fMRI can be considered for experimental design. Besides, functional near-infrared spectroscopy technology is also a good alternative in view of its portability, less cost, good temporal and spatial resolution (Scherer et al., 2012), and its feasibility in investigating resting-state or task-state FC in the human language network (Molavi et al., 2014; Huang et al., 2016). If the proposed behavioral and neural mechanisms are confirmed, new evidence will be provided for MMORPGs’ educational effect on FL/L2 learning. These new findings may promote the development of educational MMORPGs, and more importantly, pedagogical innovations can thereby be expected in the field of FL/L2 teaching.

## Author Contributions

YZ and XZ designed this study. YZ wrote this paper. HS provided suggestions on the structure of this paper. XL, DT, and Y-eC contributed to the data collection.

## Conflict of Interest Statement

The authors declare that the research was conducted in the absence of any commercial or financial relationships that could be construed as a potential conflict of interest.
