# Corrigendum: Language Learning Enhanced by Massive Multiple Online Role-Playing Games (MMORPGs) and the Underlying Behavioral and Neural Mechanisms

**DOI:** 10.3389/fnhum.2019.00307

**Published:** 2019-09-04

**Authors:** Yongjun Zhang, Hongwen Song, Xiaoming Liu, Dinghong Tang, Yue-e Chen, Xiaochu Zhang

**Affiliations:** ^1^Center for Biomedical Engineering, School of Information Science and Technology, University of Science and Technology of China, Hefei, China; ^2^School of Foreign Languages, Anhui Jianzhu University, Hefei, China; ^3^School of Humanities and Social Science, University of Science and Technology of China, Hefei, China; ^4^School of Public Affairs, University of Science and Technology of China, Hefei, China; ^5^CAS Key Laboratory of Brain Function and Disease, School of Life Science, University of Science and Technology of China, Hefei, China; ^6^State Key Laboratory of Brain and Cognitive Science, Institute of Biophysics, Chinese Academy of Sciences, Beijing, China; ^7^Center of Medical Physics and Technology, Hefei Institutes of Physical Science, Chinese Academy of Sciences, Hefei, China

**Keywords:** Massive Multiple Online Role-Playing Games (MMORPGs), language learning, interaction, reward, behavioral mechanism, neural mechanism

In the original article, we neglected to include the funder “Social Science Planning Foundation of Anhui Province, AHSKYG2017D138” to “Yongjun Zhang.”

A correction has therefore been made to the **Acknowledgement** statement:

“This work was supported by grants from the Humanities and Social Science Research Foundation of Education Department of Anhui Province (SK2015JD11), the General Project of Humanities and Social Sciences of the Ministry of Education in China (13YJC740112), and the Social Science Planning Foundation of Anhui Province, AHSKYG2017D138. We thank Yamikani Ndasauka for his suggestions during the proofreading of this paper.”

Additionally, in the published article, there was an error in affiliation “1 and 2.” Instead of “1 School of Foreign Languages, Anhui Jianzhu University, Hefei, China” and “2 Center for Biomedical Engineering, School of Information Science and Technology, University of Science and Technology of China, Hefei, China,” it should be “1 Center for Biomedical Engineering, School of Information Science and Technology, University of Science and Technology of China, Hefei, China” and “2 School of Foreign Languages, Anhui Jianzhu University, Hefei, China.”

The authors apologize for these errors and state that they do not change the scientific conclusions of the article in any way. The original article has been updated.

